# Inhibition of the integrin/FAK signaling axis and c-Myc synergistically disrupts ovarian cancer malignancy

**DOI:** 10.1038/oncsis.2016.86

**Published:** 2017-01-30

**Authors:** B Xu, J Lefringhouse, Z Liu, D West, L A Baldwin, C Ou, L Chen, D Napier, L Chaiswing, L D Brewer, D St. Clair, O Thibault, J R van Nagell, B P Zhou, R Drapkin, J-A Huang, M L Lu, F R Ueland, X H Yang

**Affiliations:** 1 Department of Pharmacology and Nutritional Science, University of Kentucky, Lexington, KY, USA; 2 Department of Molecular and Cellular Biochemistry, University of Kentucky, Lexington, KY, USA; 3 Markey Cancer Center, University of Kentucky, Lexington, KY, USA; 4 Department of Obstetrics and Gynecology, University of Kentucky, Lexington, KY, USA; 5 Department of Pathology, University of Kentucky, Lexington, KY, USA; 6 Department of Respiratory Medicine, First Affiliated Hospital of Soochow University, Jiangsu Province, PR China; 7 Department of Toxicology and Cancer Biology, University of Kentucky, Lexington, KY, USA; 8 Department of Gynecologic Cancer Research, Basser Center for BRCA, University of Pennsylvania, Perelman School of Medicine, Philadelphia, PA, USA; 9 Department of Biomedical Science, Florida Atlantic University, Boca Raton, FL, USA

## Abstract

Integrins, a family of heterodimeric receptors for extracellular matrix, are promising therapeutic targets for ovarian cancer, particularly high-grade serous-type (HGSOC), as they drive tumor cell attachment, migration, proliferation and survival by activating focal adhesion kinase (FAK)-dependent signaling. Owing to the potential off-target effects of FAK inhibitors, disruption of the integrin signaling axis remains to be a challenge. Here, we tackled this barrier by screening for inhibitors being functionally cooperative with small-molecule VS-6063, a phase II FAK inhibitor. From this screening, JQ1, a potent inhibitor of Myc oncogenic network, emerged as the most robust collaborator. Treatment with a combination of VS-6063 and JQ1 synergistically caused an arrest of tumor cells at the G2/M phase and a decrease in the XIAP-linked cell survival. Our subsequent mechanistic analyses indicate that this functional cooperation was strongly associated with the concomitant disruption of activation or expression of FAK and c-Myc as well as their downstream signaling through the PI3K/Akt pathway. In line with these observations, we detected a strong co-amplification or upregulation at genomic or protein level for FAK and c-Myc in a large portion of primary tumors in the TCGA or a local HGSOC patient cohort. Taken together, our results suggest that the integrin–FAK signaling axis and c-Myc synergistically drive cell proliferation, survival and oncogenic potential in HGSOC. As such, our study provides key genetic, functional and signaling bases for the small-molecule-based co-targeting of these two distinct oncogenic drivers as a new line of targeted therapy against human ovarian cancer.

## Introduction

Epithelial ovarian cancer, primarily high-grade serous-type ovarian cancer (HGSOC), is one of the most deadly threats to women's health worldwide. Over 70% of HGSOC patients are diagnosed at advanced and metastatic stages, and their 10-year survival rate is below 30%.^1, 2^ Also, the current treatment of ovarian cancer is largely dependent on the limited success of chemotherapeutic agents, such as paclitaxel and carboplatin, thereby being strongly associated with rapid drug resistance and poor clinical outcomes.^3^ Hence, the improvement in treatment options, including the use of target-based therapies, is urgently needed to combat the malignancy of human ovarian cancer.

Integrins, a family of heterodimeric adhesion receptors for diverse extracellular matrices, have consistently been implicated as crucial drivers of ovarian cancer development and progression.^4, 5^ Clinically, a number of the RGD-based members of the integrin family, including α5β1, and αvβ3 or αvβ5 integrins, are markedly elevated in aggressive ovarian tumors.^6, 7, 8, 9^ These adhesion receptors appear to promote cell adhesion, survival, motility and invasion during ovarian tumor growth or metastatic progression.^10, 11, 12, 13^ Importantly, the functions of these integrins are strongly dependent on the activation of focal adhesion kinase (FAK) and its downstream signaling, including the PI3K/Akt- and Ras/MAPK-dependent pathways.^5, 14, 15^ It is of no surprise that inhibition of integrin function and signaling has emerged as one of highly attractive therapeutic options for overcoming the malignancy of human ovarian cancer.

Inhibition of the FAK-dependent signaling is one of crucial means to disrupt the integrin-associated ovarian cancer malignancy.^14, 16^ As a 120 kDa intracellular non-receptor tyrosine kinase, FAK is strongly activated, once integrins are engaged with their extracellular matrix ligands, such as laminins, fibronectin and collagens.^17^ Pathologically, FAK expression is markedly elevated in a large portion of ovarian carcinomas via gene amplification or at protein level.^16^ The aberrant expression of FAK appears to be associated with drug resistance and poor clinical outcomes in ovarian cancer patients.^18, 19^ Also, the integrin/FAK-dependent signaling promotes ovarian tumor cell proliferation and survival through diverse pathways.^14, 16^ Knockdown or deletion of FAK impairs tumor growth and metastatic progression of ovarian cancer.^20, 21, 22, 23^ These growing studies support FAK inhibition as a promising avenue for mitigating ovarian cancer malignancy.

Recently, a number of small-molecule inhibitors of FAK have displayed strong clinical application potential for ovarian cancer malignancy.^18, 19, 24, 25, 26^ Notably, TAE-226 and VS-6063, two of most potent inhibitors against active FAK through disruption of its Y^397^ residue, are highly effective in inhibiting ovarian tumor growth and metastatic potential.^20, 21, 22, 23^ Such inhibition appears to be inversely associated with the expression of NF2/Merlin in carcinomas cells.^26, 27, 28^ Despite such strong antitumor effect and presence of promising biomarkers, the FAK inhibitors exhibit strong off-target effects during the clinical trial.^17^ Thus, a better understanding of the functional and signaling bases of these inhibitors will be crucial for their potential application in the clinical treatment of human ovarian cancer.

Here, we explored a strategy for enhancing the therapeutic potential of inhibiting the integrin–FAK-dependent signaling in ovarian cancer. By screening arrays of anti-cancer chemical compounds, we attempted to identify the inhibitors that were strongly cooperative with VS-6063. Also, we conducted mechanistic analyses to unravel key molecular, signaling, genetic and clinical bases underlying this cooperative effect. Results from our analyses showed that JQ1, an inhibitor of the interaction between Myc oncogenic network or their interactions with the Bromodomain-containing transcription factors (e.g., BRD4),^29, 30^ is the most robust collaborator of VS-6063. JQ1 and VS-6063 also exhibited a strong synergy in the inhibition of FAK activity and c-Myc expression, as well as their signaling through the PI3K/Akt pathway. In addition, we detected a strong concordance in the aberrant genetic changes or expression for FAK and c-Myc in the primary tumors of human HGSOC. As such, our study has identified JQ1 as a key ally of VS-6063 with respect to disruption of oncogenic processes or signaling. Our findings support the combination of these two inhibitors being a promising targeted therapy for the treatment of human HGSOC.

## Results

### Identification of JQ1 as a robust collaborator of VS-6063 by chemical inhibitor-based screening

**Figure 1:**
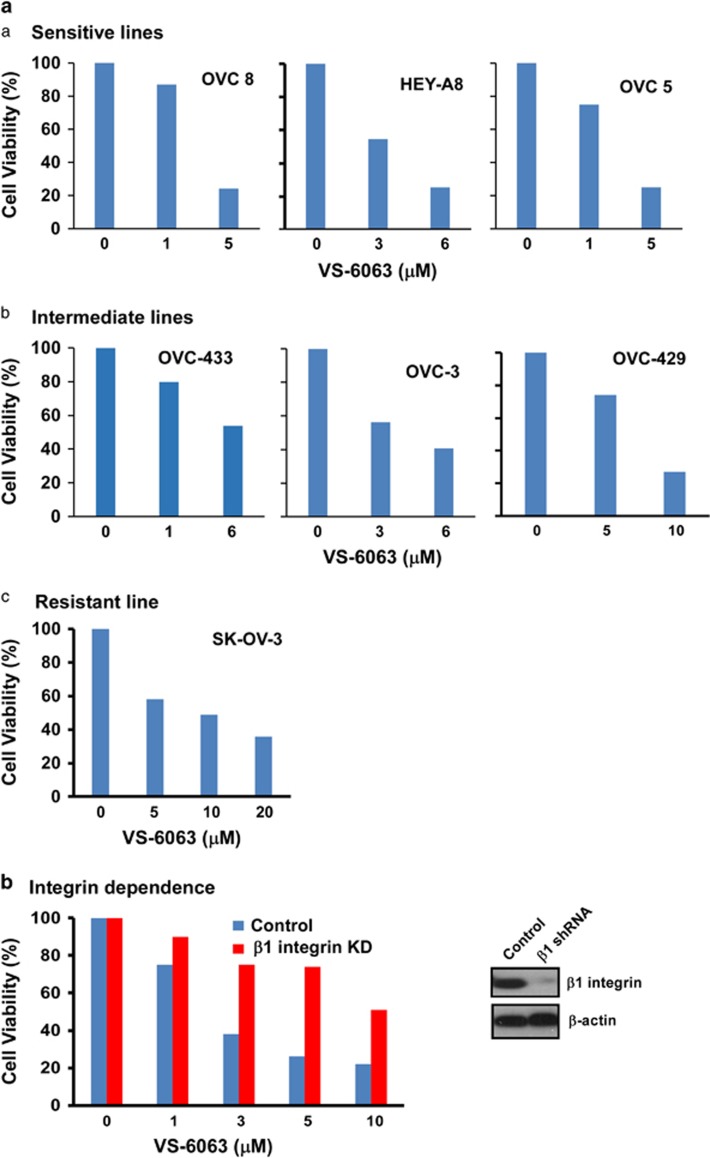
Characterization of ovarian cancer cell sensitivity to VS-6063. (**a**) Differential response of cultured human low- or high-grade ovarian cancer lines to VS-6063. Human ovarian cancer cells were grown in the presence of 5–10% fetal bovine serum, treated with indicated doses of VS-6063 for 72 h, and assessed for cell viability with the MTT assay. (a) Highly sensitive lines: OVCAR-8, OVCAR-5 and Hey-8. (b) Moderately sensitive lines: OVCAR-433, OVC-3 and OVC-429. (c) Resistant line: SK-OV-3. (**b**) Evaluation of the β1 integrin dependence of VS-6063. OVCAR-5 cells with (blue bar)or without β1 integrin stable knockdown (red bar) were treated with varying doses of VS-6063 for 72 h, followed by analysis of viability. The extent of β1 integrin knockdown was confirmed by immunoblotting (right panel). All data are representative of one of three independent experiments. s.e. (standard errors of the means): <5% (not shown). Values: percentages of the viability of treated cells over control—0.1% DMSO, *n*=3.

As a crucial driver of ovarian cancer malignancy, the integrin–FAK signaling axis remains a challenge regarding its therapeutic targeting, largely because of the potential off-target effect of FAK inhibitors.^17^ To overcome this clinical application barrier, we sought to screen for a set of small-molecule inhibitors being cooperative or synergistic to VS-6063, a phase II inhibitor of active FAK, which is characterized by the phosphorylation at Y^397^ residue.^28^ To construct our cell viability-based screening system, representative human HGSOC cell lines were analyzed for their sensitivities to VS-6063. As shown in Figure 1a, OVC-8, OVC-5 and HeyA8 lines were most sensitive to VS-6063, while OVC-429, OVC-433 and OVC-3 lines exhibited intermediate or moderate response. In comparison, SK-OV-3, a low-grade ovarian tumor line, had the least sensitivity (Figure 1c), consistent with a prior study.^26^ Furthermore, the specific effect of VS-6063 on the integrin-induced FAK activation was also validated, as OVC-5 cells exhibited a marked decrease in the sensitivity to this inhibitor upon the stable knockdown of β1 integrin (Figure 1b). Based on these observations, OVC-433 line was employed as an *in vitro* model for our subsequent screening for small-molecule inhibitors being functionally cooperative to VS-6063.

**Figure 2:**
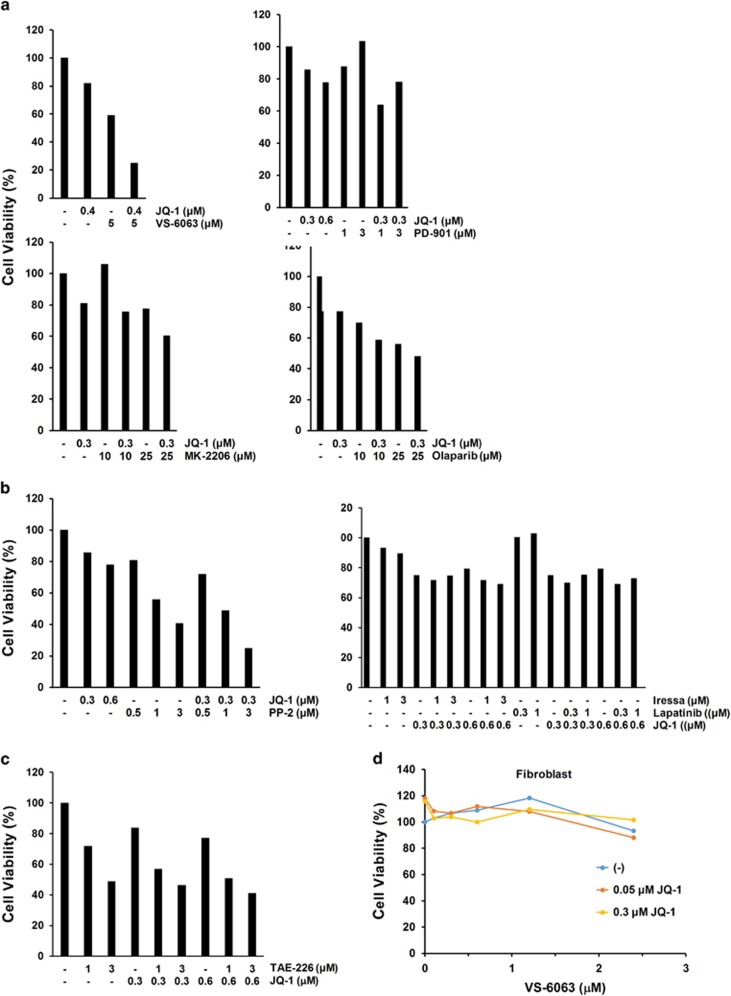
Identification of JQ1 as a collaborator of VS-6063 by using chemical inhibitor-based screening. (**a**–**c**) Evaluation of the inhibitory effect of VS-6063 or TAE-226 and candidate inhibitors on the viability of OVC-433 cells. (**d**) Evaluation of the potential off-target effect of JQ1 and VS-6063 combination in primary human fibroblasts. The functional evaluation of inhibitors was conducted with OVC-433 cells over a period of 72 h in the presence of 10% fetal bovine serum. The cell viability was measured by the MTT assay. Values: percentages of viable cells over the dimethyl sulfoxide control (*n*=2). All s.e.'s were <5% of the mean.

To seek functional collaborators for the integrin–FAK signaling axis, we evaluated the synergistic or cooperative effects of VS-6063 with 30 potent inhibitors against diverse oncogenic pathways in human cancer (Supplementary Table S2). Data from these analyses revealed that JQ1, a newly identified inhibitor of Myc oncogenic network, exhibited the most synergistic cooperation with VS-6063 in terms of inhibiting ovarian cancer cell viability (Figure 2a). By comparison, there was a minimal synergy for other inhibitors of prominent oncogenic pathways, including MK-2206 for PI3K/Akt, PD-901 for RAS/MAPK, Olaparib for PARP involved in the DNA repairing and ICG001 for the canonical Wnt signaling (data not shown). The functional cooperation between VS-6063 and JQ1 also appeared to be highly specific, as JQ1 exhibited less or minimal cooperation with other tyrosine kinase inhibitors, such as PP2 (an inhibitor of Src family tyrosine kinases) or Iressa/Lapatinib (inhibitor of EGFR or ErbB2) (Figure 2b). Also, I-BET151, which is functionally similar to JQ1,^31^ exhibited a strong synergy with VS-6063 (data not shown). However, JQ1 exhibited very little synergy with TAE-226 (Figures 2b and c), an inhibitor being functionally similar to VS-6063.^25^ Thus, these data support JQ1 as a strong collaborator of VS-6063 in terms of disruption of ovarian cancer cell viability.

**Figure 3:**
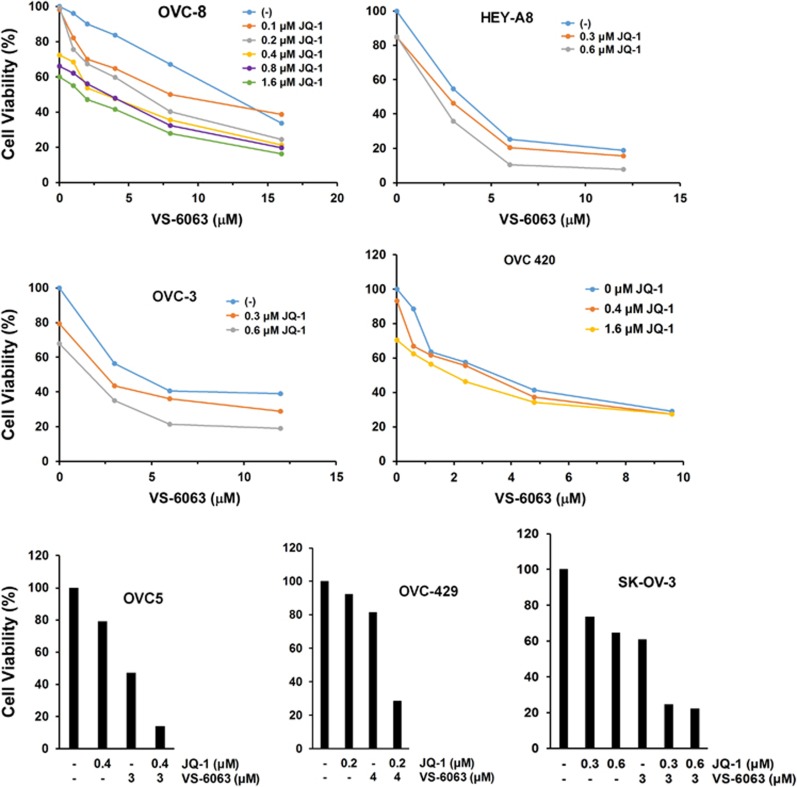
Defining the scope of the effectiveness of VS-6063 and JQ1 combination. A total of six different HGSOC cell lines (OVC-8, Hey-8, OVC-3, OVC-5, OVC-420 and OVC-429) and one low-grade (SK-OV-3) cell lines were treated with indicated inhibitors for 72hrs, followed by analysis of cell viability with MTT assay. Values: percentages of viable cells over the dimethyl sulfoxide control (*n*=2). All s.e.'s were <5% of the mean.

Moreover, we observed a minimal cooperative impact of VS-6063 and JQ1 on the viability of normal human fibroblasts (Figure 2c), suggesting a minimal toxic or off-target effect. In line with this notion, the synergistic or additive effect of VS-6063 and JQ1 was detected across a panel of human HGSOC cell lines except OVC-420 (Figure 3), regardless their differential sensitivities to VS-6063 (Figure 1). Interestingly, our extensive analyses showed that low doses of JQ1 (0.1–1.6 μm) appeared sufficient to enhance the inhibitory effect of VS-6063 in multiple ovarian cancer cell lines (Figure 3), implicating a strong catalyst-like role of JQ1. Taken together, these observations indicate that JQ1 likely acts as a catalyst for the inhibitory effect of VS-6063 on the FAK-driven cell viability in ovarian cancer.

### Scope of functional cooperation of VS-6063 and JQ1

**Figure 4:**
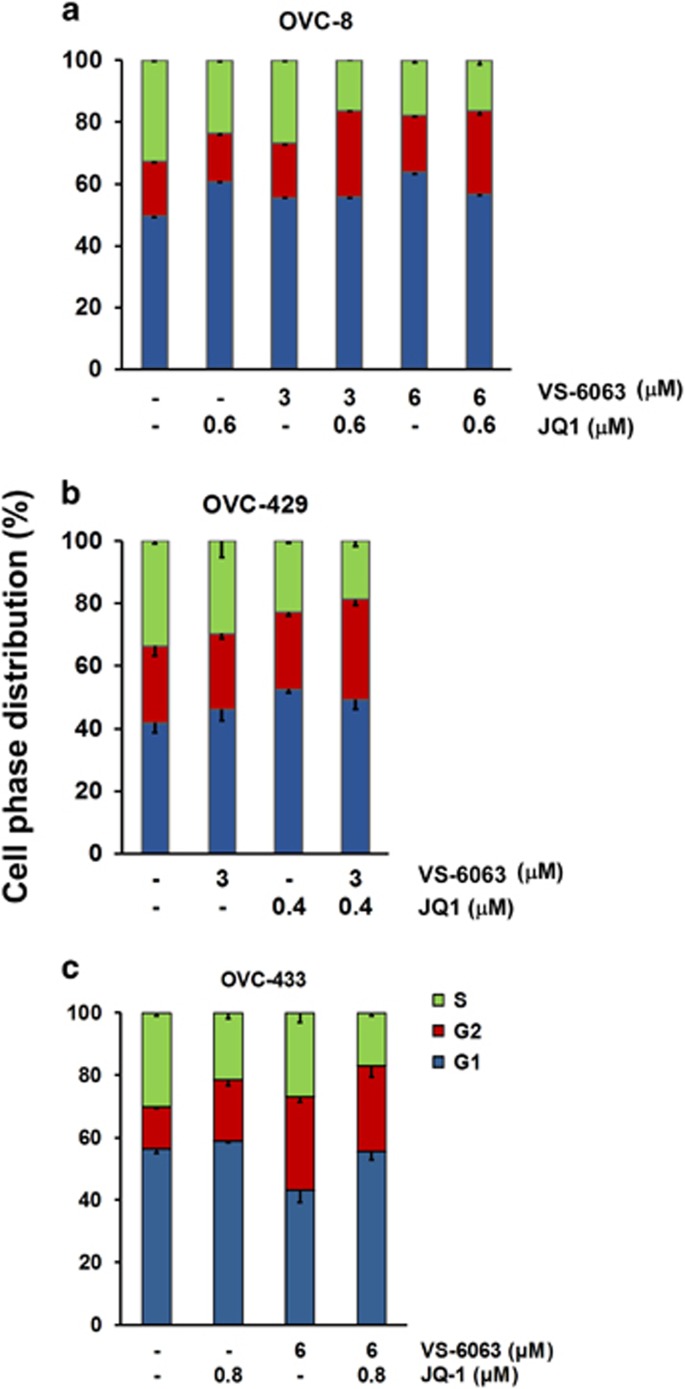
Effect of VS-6063 and JQ1 combination on cell cycle progression. OVC-8 (**a**), OVC-429 (**b**) and OVCAR-433 (**c**) cells were cultured in the presence of dimethyl sulfoxide or inhibitors for 48 h and followed by analyses of PI staining on flow cytometry. Values: percentage of cells at the cell phases indicated (mean±s.e., *n*=3). Data was representative of three independent experiments.

**Figure 5:**
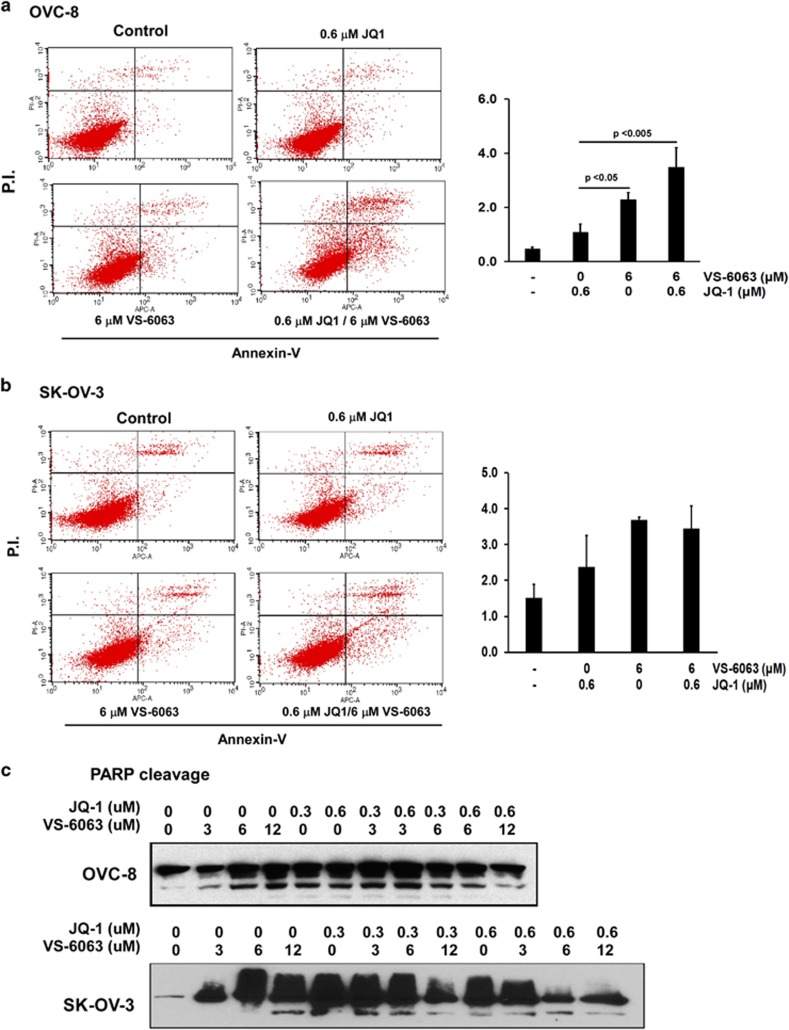
Effect of VS-6063 and JQ1 on ovarian tumor cell survival. (**a**, **b**) Flow cytometry-based analyses of Annexin V and PI-positive cells in response to treatment of VS-6063 and JQ1. OVC-8 or OVC-433 cells were treated with inhibitors over 48 h, detached and stained with propidium iodide and APC-conjugated Annexin V, and quantified by flow cytometry (left panels). Values (right panels): Mean±s.e., *n*=3. (**c**) Analyses of PARP1 cleavages in tumor cells treated with varying doses of VS-6063 and JQ1. Tumor cells were lysed in RIPA buffer, and immunoblotted with antibody against PARP1 or β-actin (as a control).

We further investigated if the functional cooperation of VS-6063 and JQ1 in ovarian cancer extended beyond cell viability. Our analyses showed that the combination of VS-6063 and JQ1 led to a significant decrease in the portion of cells entering into the S phase, but an increase in cell arrest at G2/M in all three cancer cell lines (OVC-429, OVC-8 and OVC-433), compared with the use of individual inhibitors alone (Figure 4). Furthermore, a synergistic or additive effect of this inhibitor combination was detected regarding the survival of VS-6063-sensitive OVC-8 cells, but not SK-OV-3 cells (Figures 5a and b). Interestingly, JQ1 at the dose range of 0.3–0.6 μm also exhibited a strong synergy with VS-6063 in induction of cell apoptosis, as reflected by an increased cleavage of PARP proteins in SK-OV-3 cells, but not in OVC-8 cells (Figure 5c). In line with these observations, we detected a strong synergy of VS-6063 and JQ1 with respect to the disruption of cell morphologies (Supplementary Figure S1). These observations implicated a broad functional collaboration for VS-6063 and JQ1 in ovarian cancer, particularly regarding cell proliferation and survival.

### Molecular and signaling bases of functional cooperation between VS-6063 and JQ1

**Figure 6:**
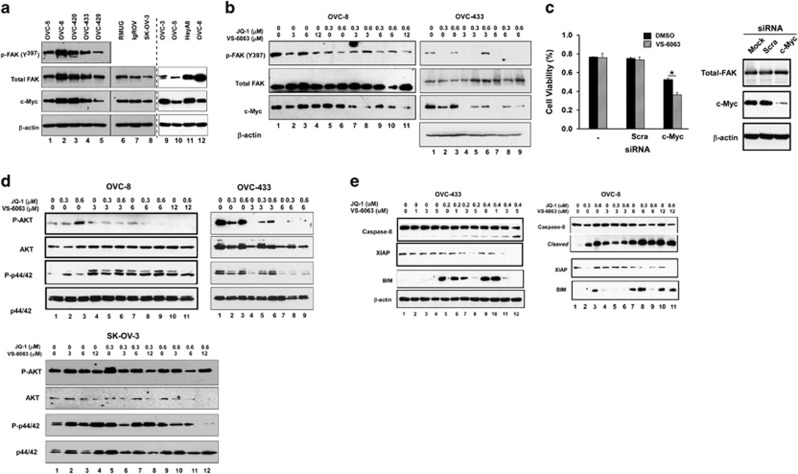
Identification of the key targets and pathways of VS-6063 and JQ1 in ovarian tumor cells. (**a**) Expression of total or phosphorylated FAK or Myc in human ovarian tumor cell lines. Blotting for lanes 1–8 and lanes 9–12 was conducted separately with the lysates from the indicated human HGSOC cell lines. (**b**) OVC-8 or OVC-433 cell lines were treated with indicated inhibitors for 48 h, followed by analyses of levels of total or phosphorylated FAK or Myc proteins. Equal protein loading for OVC-8 cells was also assessed by blotting β-actin shown in Figure 5c. (**c**) OVC-8 cells were treated with siRNA for 24 h, and followed by incubation with 2 μm VS-6063 for additional 48 h. Tumor cells were then analyzed by the MTT assay or blotted for c-Myc. Values (right panels): mean±s.e., *n*=3. *P-value <0.05. (**d**) Effect of VS-6063 and JQ1 on key downstream pathways of the integrin–FAK signaling axis. OVC-8, OVC-433 and SK-OV-3 cells were treated with different combinations or dosages of VS-6063 and JQ1 alone or in combination for 48 h and analyzed for the activation of the PI3k/AKT or Ras/MAPK pathway. (**e**) Effect on the cell survival-related pathway. Tumor cells treated with various doses of inhibitors for 48 h and analyzed for differences in cleaved Caspase 8, XIAP and Bim by immunoblotting. β-Actin was blotted as a control for OVC-433 or OVC-8 (shown in Figure 5c).

Next, we attempted to delineate the molecular and signaling bases underlying the functional cooperation of VS-6063 and JQ1 in ovarian cancer. We noted that expression of FAK and c-Myc, which are regarded as key targets of VS-6063 and JQ1, respectively,^28, 30^ varied among the cultured HGSOC cell lines (Figure 6a). In particular, the VS-6063-resistant SK-OV-3 line had low expression of active or total FAK or c-Myc. (Figures 1a and 6a). By comparison, there was a lack of an apparent association for BRD4, another key target of JQ1 (data not shown). Interestingly, the varying expression of FAK and c-Myc in ovarian cancer cell lines appeared to be closely correlated with their responses to the treatment of these two inhibitors. Notably, both OVC-8 and OVC-433 lines displayed a dose-dependent decrease in the phosphorylation of FAK or c-Myc protein upon the treatment with VS-6063 or JQ1 or combination (Figures 6a and b). Consistent with these observations, c-Myc knockdown decreased tumor cell sensitivity to the VS-6063 treatment (Figure 6c). Together, our data illustrate that ovarian tumor cell response to the treatment of VS-6063 and c-Myc is strongly dependent on the activation or expression of FAK and c-Myc.

The strong cooperative effect of VS-6063 and JQ1 on the activation of FAK and/or Myc was further reflected by a corresponding decrease in their signaling through the PI3K/Akt in OVC-8 and OVC-433 lines (Figure 6d). In comparison, there was a limited or minimal difference in the Ras/MAPK pathway. Furthermore, our analyses showed that these two inhibitors synergistically inhibited the survival of ovarian tumor cells via activation of the Caspase 8/XIAP-dependent pathway (Figure 6e). However, such inhibitor combination appeared to have a minimal impact on the activation of the PI3K/Akt or Erk1/2 pathway in VS-6063-resistant SK-OV-3 line. Taken together, our results demonstrate that the synergistic role of VS-6063 and JQ1 in ovarian cancer is attributed to their impact on the activation of FAK and c-Myc, as well as their downstream signaling.

### Genetic bases for the simultaneous disruption of FAK and c-Myc

**Figure 7:**
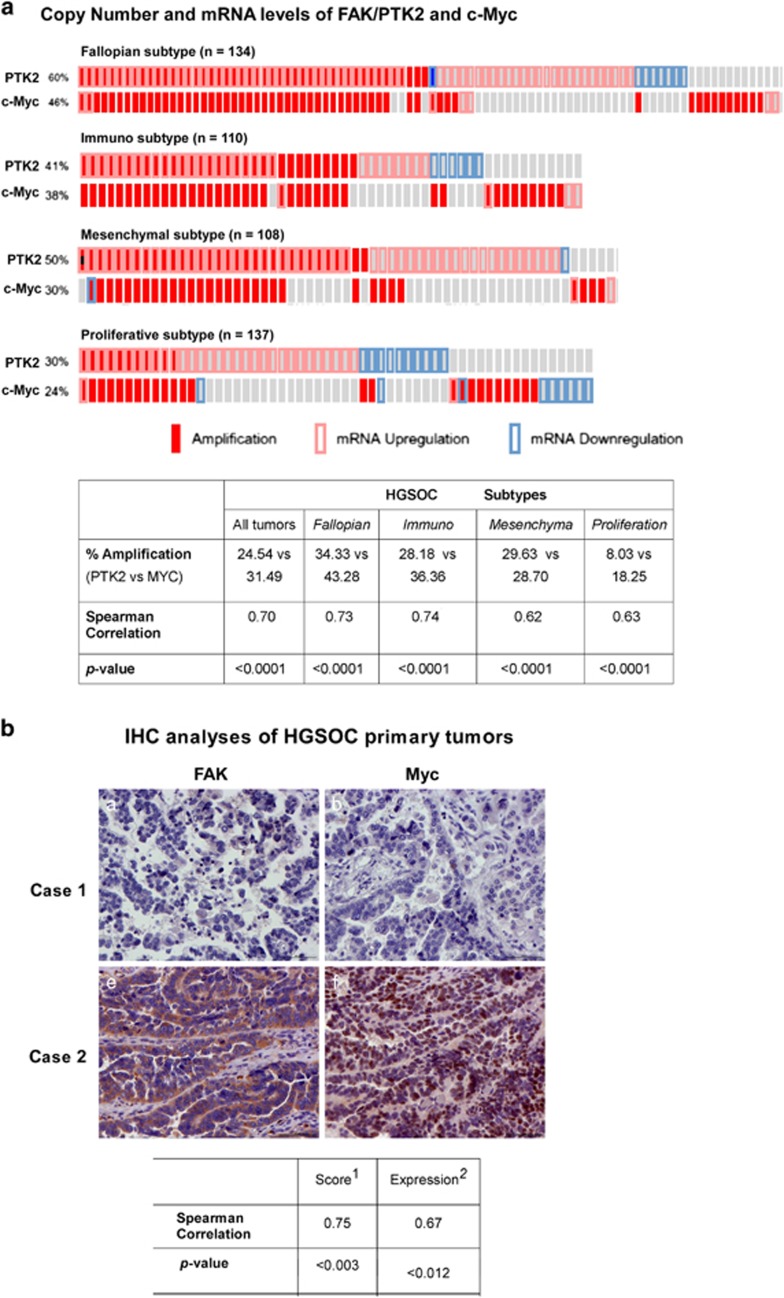
Gene amplification or aberrant expression of FAK and c-MYC in human HGSOC primary tumors. (**a**) Co-amplification of FAK/PTK2 and c-Myc in HGSOC. Top panel: Differential upregulation of FAK in the primary tumors of four distinct subtypes in the TCGA cohort of HGSOC patients. Bottom panel: Statistical evaluation of the co-amplification of FAK and Myc in primary tumors among HGSOC subtypes from the TCGA patient cohort. (**b**) Co-upregulation of FAK and c-MYC in primary tumors of local HGSOC patient cohort (*n*=15). Top panel: Representative images of FAK and Myc expression from IHC analyses of consecutive sections of primary tumors. Bottom panel: Statistical assessment of the concordance of PTK2 and Myc upregulation. Scale bar: 100 μm.

Our further analyses provided genetic support for the co-targeting of the integrin–FAK signaling pathway and Myc oncogenic network in ovarian cancer. It is known that both FAK and Myc are closely located at 8q24 locus, one of the most amplified regions in ovarian tumor genomes.^32^ Thus, it was of no surprise that FAK and c-Myc exhibited extensive co-amplification in HGSOC primary tumors in the TCGA patient cohort (Figure 7a). Such co-amplification appeared to be independent of tumor subtypes. By comparison, FAK displayed a minimal co-amplification with N-Myc or BRD4 (data not shown), which are also implicated as crucial targets of JQ1.^30^ In addition, we observed a strong co-upregulation of FAK and c-Myc in the primary tumors of a local HGSOC patient cohort (*P*<0.03) (Figure 7b). Together, these results unveil genetic and clinical bases underlying simultaneous alterations in FAK and c-Myc in ovarian cancer.

### Modulation of tumor cell sensitivity to chemotherapeutic agents by VS-6063 and JQ1

**Figure 8:**
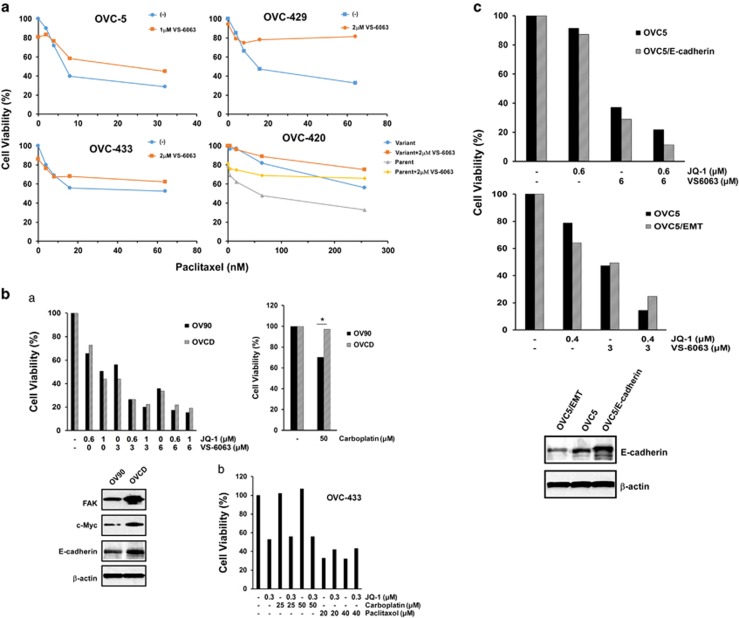
A link of VS-6063 and JQ1 to the drug resistance and EMT trait in ovarian cancer. (**a**) Effect of VS-6063 on tumor cell response to paclitaxel in human HGSOC cell lines, including OVC-5, OVC-429 and OVC-433, as well as OVC-420 and its variant. (**b**) Effect of VS-6063 or JQ1 or both on tumor cell response to carboplatin in OV-90 and its carboplatin-resistant variant (OVCD), as well as OVC-433. (**c**) Effect of EMT on tumor cell response to the treatment of VS-6063 and JQ1. Analyses were performed with OVC-5 parent and its E-cadherin-overexpressing OVC-5 cells (prepared by transfection and sorting on flow cytometry), along with a spontaneous EMT variant developed during cell culture. Values: percentages of viable cells over the dimethyl sulfoxide control (*n*=3). All s.e.'s were <5% of the mean. The MTT assay was conducted after tumor cells being treated for 48 hrs (**a**) or 72 hrs (**b**, **c**).

Based on the studies of the integrin–FAK signaling axis,^19, 33^ we also evaluated the effect of the VS-6063 and JQ1 combination on the chemoresistance of ovarian cancer. Our data showed that in multiple ovarian cancer cell lines, treatment with a low concentration of VS-6063 alone actually led to more resistance to paclitaxel in ovarian cancer cells (Figure 8a). In contrast, treatment with VS-6063 and JQ1 combination appeared to be equally effective in the inhibition of viability of parent and carboplatin-resistant OV-90 cells (Figures 1 and 8). Together, these data indicate the effectiveness of VS-6063 and JQ1 combination in ovarian cancer likely goes beyond tumor chemoresistance.

Since activation of the integrin–FAK signaling axis is frequently upregulated in the tumor cells conferring the epithelial–mesenchymal transition (EMT) phenotype,^17^ we examined the role of VS-6063 and JQ1 combination in the EMT-associated chemoresistance. As shown in Figure 8c, the EMT variant of OVC-5 line exhibited more resistance to the treatment of VS-6063 and JQ1 combination, compared with their counterparts (*p*<0.05). Conversely, the forced expression of E-cadherin reversed this effect (*p*<0.05). These observations indicate that treatment with VS-6063 and JQ1 combination is effective in impairing the EMT-associated tumor cell viability in ovarian cancer.

## Discussion

While the integrin–FAK signaling axis and Myc oncogenic network are both implicated in promoting HGSOC tumor development and progression,^17, 30, 34^ a simultaneous targeting of these two key drivers has rarely been considered as a targeted therapy for the treatment of such deadly disease. Here, we reported a strong synergy between JQ1, a newly identified potent inhibitor of Myc oncogenic network, and VS-6063, a small molecule blocking the activation of integrin-mediated FAK, in terms of the inhibition of ovarian tumor cell proliferation and survival. Our data also reveal that the functional cooperation of VS-6063 and JQ1 is linked to their impact on the action of FAK (phosphorylated at Y^397^ residue) and Myc, as well as their signaling through the PI3K/Akt pathway. In line with such observation, the siRNA-mediated Myc downregulation impairs ovarian tumor cell sensitivity to VS-6063. Treatment with a combination of VS-6063 and JQ1 also effectively decreases intrinsic or the EMT-associated chemoresistance of ovarian cancer cells. Clinically, Myc and FAK appear to be co-amplified or co-upregulated at the genomic or protein level in the primary tumors of human HGSOC. Thus, our study provides strong molecular, cellular and genetic bases for the co-targeting of the integrin–FAK signaling axis and Myc to mitigate ovarian cancer malignancy.

Our study suggests that the synergistic or cooperative effect of VS-6063 and JQ1 is largely attributed to the inhibition of ovarian cancer cell survival (Figures 4 and 5). Mechanistically, this appears to be linked to the effect on the XIAP/Bim-mediated and caspase 8-dependent apoptosis (Figure 6a). While Bim is long recognized for its antagonizing role in the integrin-mediated tumor cell survival,^35^ it is strongly induced by JQ1, according to recent studies.^36^ Thus, VS-6063 and JQ1 may synergistically inhibit ovarian tumor cell survival largely by mitigating the integrin–FAK-dependent cell survival or activation of the Bim/caspase 8-linked apoptotic process. It is also worth noting that the marked synergistic effect of VS-6063 and JQ1 on the G_2_/M transition of ovarian cancer cells is consistent with the strong role of the integrin–FAK signaling axis and the Myc oncogenic network in cell proliferation. In fact, both VS-6063 and JQ1 are independently implicated in suppressing tumor cell cycle in several human cancer types.^17, 37^ Thus, in a broad sense, the synergistic effect of VS-6063 and JQ1 on ovarian cancer are largely attributed to the combined disruption of tumor cell proliferation and survival.

One of the unexpected observations from our current study is the synergistic effect of VS-6063 and JQ1 on the activation or expression of FAK and c-Myc in ovarian cancer cells (Figure 6). Accordingly, the integrin–FAK signaling axis may drive ovarian tumorigenesis through affecting Myc activation in a BRD4-independent manner.^30^ However, such functional link may also be associated with a potential translational activation of c-Myc by FAK.^38^ In this regard, it is of no surprise that FAK is not present in the list of candidate kinase drivers obtained from a large-scale shRNA-mediated functional screening in ovarian cancer cells.^30^ Meanwhile, the synergistic role of VS-6063 and JQ1 in the inhibition of ovarian cancer cell survival or proliferation is supported by their strong effects on the signaling through the PI3K/Akt pathway, not on the Ras/MAPK pathway (Figure 6). These observations are also consistent with the well-recognized signaling or functional role of FAK or Myc across a spectrum of human cancer.^17, 39^ However, it is still intriguing that neither of these pathways appears to be affected by VS-6063 or JQ1 in the VS-6063-resistant SK-OV-3 cells (Figure 7), despite a strong effect on cell viability. Thus, future studies will be directed to understand how JQ1 modulates the VS-6063 resistance in ovarian cancer at molecular levels.

Despite the high genetic variability, the majority of HGSOC cell lines tested in the current study exhibit a robust synergistic response to the combined treatment of VS-6063 and JQ1 (Figures 1, 2, 3). Part of such synergy appears to be associated with the co-amplification or co-upregulation of FAK and Myc (OVC-8, OVC-433 and HeyA8). In contrast, the high sensitivity of OVCAR-5 cells appears to be more related to the K-Ras mutation and strong activation of MAPK-mediated signaling (Figure 3). In fact, the oncogenic activity of mutated K-Ras, particularly regarding its promotion of tumor cell survival, has been shown to be strongly dependent on the integrin–FAK signaling.^40^ In this regard, our study argues that the VS-6063-resistance of SK-OV-3 cells described in prior studies^26^ is likely attributed to the strong activation of the Ras/MAPK pathway resulted from the ErbB2 amplification (Figure 6), rather than the presence of intact Merlin/NF2.^26, 28^ Moreover, our data suggest that the functional cooperation of VS-6063 and JQ1 is minimally affected by the status of p53 or BRCA1/2-associated DNA repairing pathways. Notably, OVC-420 cells are responsive to the combined inhibition of these two inhibitors, despite of expression of intact p53. Also, OVC-433 cells are responsive to the treatment of the VS-6063 and JQ1 combination, despite carrying deregulated BRCA1/2.^41^ Collectively, our study supports the notion that the treatment of VS-6063 and JQ1 combination should be effective against human HGSOC across a broad oncogenic spectrum.

Our current study suggests that disrupting the integrin–FAK signaling has a limited impact on the paclitaxel resistance of ovarian cancer, consistent with a prior study.^42^ Yet, this observation differs from a recent analysis with an FAK inhibitor.^19^ The precise reason behind such discrepancy currently remains unclear. Meanwhile, if our results hold, the combination of VS-6063 and JQ1, instead of FAK inhibitor alone, should serve as an effective target therapy to overcome the drug resistance-associated malignancy in HGSOC. Given the dose-related toxicity or side effect for VS-6063 or other FAK inhibitors (e.g., TAE-226), the inclusion of JQ1-type inhibitor should lower the IC_50_ of FAK inhibitors, thereby enhancing the effectiveness of VS-6063-based targeting in ovarian cancer.

It is also worth noting that the VS-6063 and JQ1 inhibitors may have a synergistic or cooperative effect on ovarian tumor progression or metastasis, despite not being evaluated in the current study. In fact, the integrin–FAK signaling axis has been consistently linked to cell motility, invasion and colonization during ovarian cancer metastasis.^4^ Also, JQ1 confers an inhibitory role in tumor cell invasion.^43^ In line with this notion, a number of HGSOC cell lines that confer strong invasive potential and EMT phenotype, including OVCAR-8, OVCAR-433 and OVCAR-5, appear to express high levels of active FAK and Myc (Figures 6 and 8). These lines are also highly susceptible to the synergistic inhibition of VS-6063 and JQ1 in terms of tumorsphere formation (Supplementary Figure S2). In addition, JQ1 is implicated in suppressing the activation of Twist1, a strong driver of cell invasion and extracellular matrix remodeling in metastatic ovarian cancer.^43, 44^ Collectively, the treatment with the VS-6063 and JQ1 combination appears to be not only effective in disrupting tumor cell survival but also have a broad functional impact, including the effect on tumor cell invasion or metastatic progression.

The frequent co-upregulation of FAK and c-Myc in ovarian tumors (Figure 7) also underscores the therapeutic potential of the VS-6063 and JQ1 combination. Since ovarian tumor cells with the co-upregulation of FAK and Myc are more sensitive to the combination of VS-603 and JQ1 than their counterparts (Figure 7), the aberrant expression of FAK and Myc likely serves as promising biomarkers for identifying the corresponding patient cohort in the clinic. Furthermore, such patient cohort is detectable through immunohistochemistry (IHC) or genomic analyses of patient tumor biopsies for co-upregulation of FAK and Myc proteins. In addition, part of such patient cohort is identifiable through molecular analyses of chromosome 8q24 locus deregulation or K-Ras mutations, according to our current study and prior analyses.^45^ Consequently, the availability of potential biomarkers and key oncogenic identities makes the combination of VS-6063 and JQ1 as one of highly promising target therapies for subsequent clinical trial on HGSOC patients.

The synergistic effect of co-targeting of FAK and c-Myc detected by the current study is also reminiscent of the synthetic lethal therapeutic strategy described for human tumors expressing highly level of c-Myc or being addicted to such oncogene.^39, 46^ This strategy is largely based on the notion that the c-Myc-driven tumorigenesis is vulnerable to the disruption of the cyclin-dependent kinases.^47^ In the current study, we also detects a more robust inhibition of tumor cell viability by the FAK inhibitor in the cell lines having high level of c-Myc (OVC-8 and HreyA8) or K-ras mutation (OVC-5) (Figures 1 and 3). Thus, part of the VS-6063 effect observed in the current study is likely attributed to its synthetic lethal effect on ovarian cancer cells. As such, our current study supports the FAK inhibitor alone as a targeted therapy for a fraction of HGSOC patients with the tumors expressing high level of c-Myc or mutated K-ras.

In summary, our study has revealed that VS-6063 and JQ1 synergistically or cooperatively disrupt tumor cell proliferation and survival in human HGSOC by inhibiting active FAK and c-Myc, as well as their signaling through the PI3K/Akt pathway. The combination of these inhibitors is selectively effective against chemoresistant ovarian tumor cells. Importantly, FAK and Myc, are not only co-amplified or upregulated in a large fraction of human HGSOC tumors but also serve as strong candidate biomarkers for identifying a corresponding patient cohort in the clinic. Consequently, the combination of VS-6063 and JQ1 is readily applied in a clinical trial as a candidate therapy for the HGSOC patients. Our study has therefore supported the combination of VS-6063 and JQ1 as a new line of targeted therapy against ovarian cancer malignancy.

## Materials and methods

### Cell lines, antibodies and chemical inhibitors

Human ovarian cancer cells lines, including HeyA8, OVCAR-3/OVC-3, OVCAR-5/OVC-5, OVCAR-8/OVC-8, OVCAR-420/OVC-420, OVCAR-429/OVC-429, OVCAR-433/OVC-433, RMUG, IGROV and SK-OV-3 cells were obtained from ATCC (Manassas, VA, USA), and authenticated by DNS finger print as described in prior studies.^12, 48^ The chemoresistant variant of OV-90 was described in a prior study.^49^ All cells lines were cultured in RPMI 1604 (Invitrogen, Carlsbad, CA, USA) supplemented with 5–10% fetal bovine serum (Sigma-Aldrich, St Louis, MO, USA) at 37 °C and 5% CO_2_. In addition, normal human fibroblasts were purchased from Lonza (Basel, Switzerland), and cultured by following the protocol provided by the vendor.

The antibodies used for IHC or immunoblotting are listed in Supplementary Table S1. The sources of small-molecule inhibitors being screened are described in Supplementary Table S2.

### Expression of SiRNA and shRNA, and generation of stable cell line

The Myc siRNA was obtained from Cell Signaling Technology (Danvers, MA, USA). For transfection, tumor cells were seeded in 24 wells and transfected with siRNA oligos plus Lipofectamine 2000 (Thermo-Fisher, Waltham, MA, USA). The transfection medium was replaced next day with fresh growth medium. After additional 72 h of culture, cells were analyzed for cell viability or lysed for verification of protein knockdown by immunoblotting.

The stable knockdown of β1 integrin in OVCAR-5 cells was conducted by viral infection with the pLKO lentiviral vector containing β1 integrin shRNA oligo, followed by subsequent two-way cell sorting on flow cytometry with a combination of GFP and β1 integrin mAb (P5D2 clone; BioLegend, San Diego, CA, USA), as described in our recent study.^50^

### Analyses of cell viability, cycle progression, survival/apoptosis and tumorsphere formation

Analyses of cell viability were conducted by the MTT assay as described in the prior studies.^51^ In brief, tumor cells were seeded into 96- or 48-well plates, and cultured overnight. In the following day, tumor cells were treated with dimethyl sulfoxide control (<0.1%) or varying types or dosages of single or multiple inhibitors for a period of 48 or 72 h, followed by analyses with the MTT assay. The values were calculated as mean % viable cells and variations between replicates were <5% of the means.

Cell cycle was measured by analyses of cells being stained with propidium iodide. In brief, tumor cells were seeded into six-well plates at 4–61 × 10^4^ per well. After being treated with dimethyl sulfoxide control or varying doses of inhibitors for 48–72 h, tumor cells were fixed in ice-cold 70% ethanol, and stored at −20 C. The fixed cells were then subjected to DNAase-free RNAase treatment for 1 h and followed by incubation with 0.5 mg/ml propidium iodide at RT for 1 h. The percentages of cell phases in the treated cell populations were subsequently analyzed by flow cytometry and quantified by use of Flow Jo software.^51^

Cell survival/apoptosis was assessed by determining the proportion of tumor cells being stained with a combination of APC-conjugated Annexin V (10 μg/ml; BioLegend) and propidium iodide (10 μg/ml), followed by analyses on flow cytometry at the core facility at UK medical center. The percentages of Annexin V-positive cells were quantified by FACS analyses on flow cytometry.

The assay of tumorsphere formation was performed as described in our prior studies.^28, 52^ In brief, 5 × 10^3^ cells were seeded in 24-well ultra-low adhesion plate, and cultured in the stem cell medium over 6 days, and imaged. Sizes of tumorspheres were quantified by use of Element software (Nikon, Melville, NY, USA).

### Signaling analyses and immunoblotting

For signaling analyses, tumor cells were starved in a medium supplemented with 0.5–1.0% fetal bovine serum overnight and followed by culturing with growth medium (10% fetal bovine serum) in the presence or absence of chemical inhibitors for additional 24 or 48 h. Tumor cells were lysed in RIPA buffer plus inhibitors.^51^ Immunoblotting of proteins or signaling molecules was conducted by incubating cell lysates with indicated primary and secondary antibodies, and detecting with the Supersignal West Pico Chemoluminescence Kit (Thermo-Fisher) The treatment of tumor cells with inhibitors or siRNA oligos were conducted as described in a prior study.^51^

### IHC analyses of paraffin-embedded human tumor tissues

The primary ovarian tumors came from the patient cohort being recently diagnosed or treated at the University of Kentucky-Chandler Medical Center. IHC staining of tumor sections was conducted according to the protocol described in a prior study.^51^ The paraffin-embedded human HGSOC tumors were obtained through Biospecimen core at the University of Kentucky.

### Statistical analyses

All data represented the results from three independent experiments. The statistical differences between groups were determined using a two-tailed Student's *t*-test or a two-tailed Mann–Whitney test. For IHC analyses of human tumor tissues, Spearman's correlation coefficient was employed to quantify correlations between Myc, total and phosphorylated FAK and tumor stage or subtypes.
