# Supplementary figures and images for: Inhibition of the integrin/FAK signaling axis and c-Myc synergistically disrupts ovarian cancer malignancy
Source: Oncogenesis. 2017 Jan 30;6(1):e295–. doi: 10.1038/oncsis.2016.86 (PMC5294249; doi:10.1038/oncsis.2016.86)

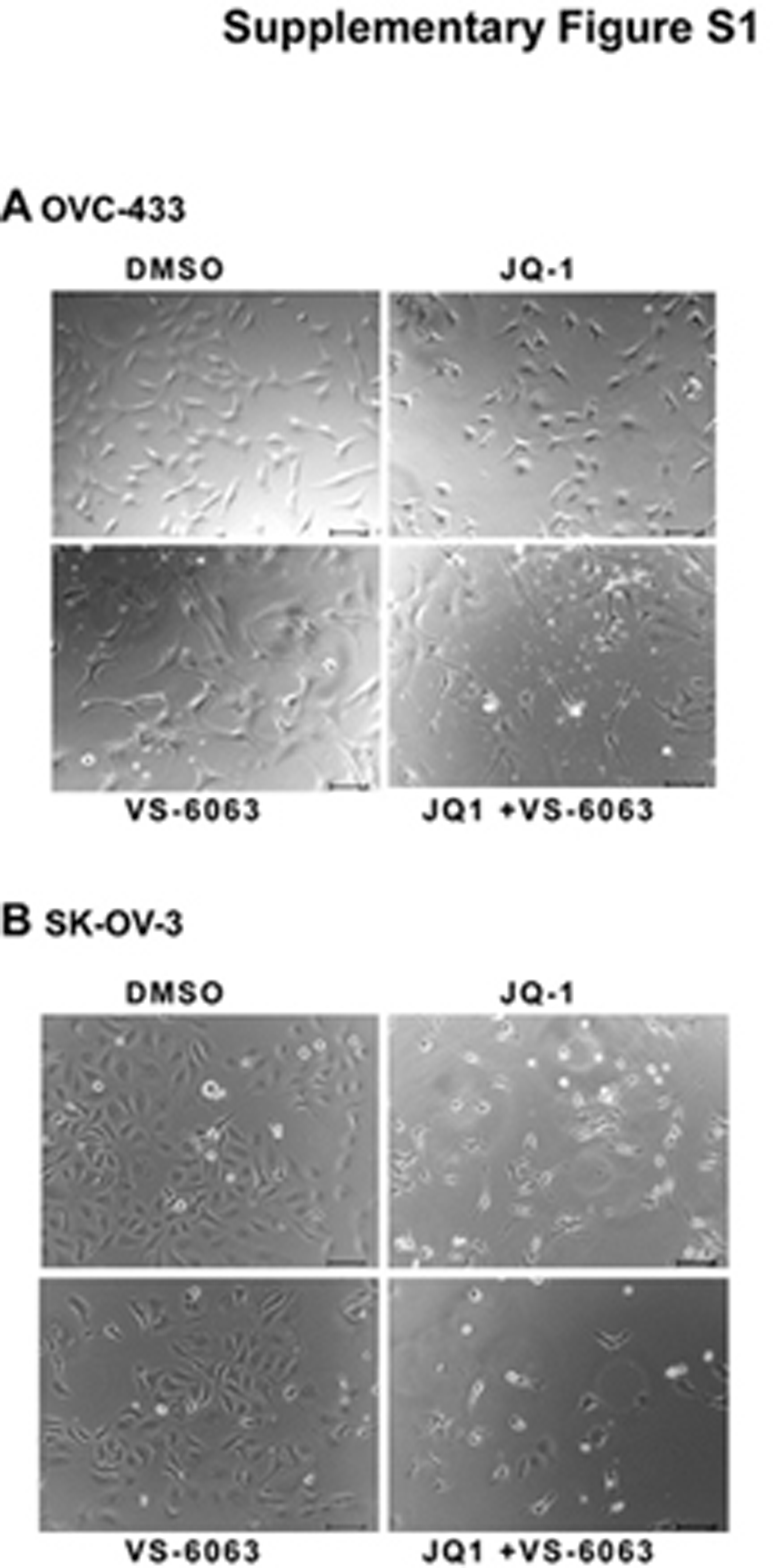

Supplement: Supplementary Figure S1 [file oncsis201686x1.tif]
