# Supplementary material for: Inhibition of the integrin/FAK signaling axis and c-Myc synergistically disrupts ovarian cancer malignancy
Source: Oncogenesis. 2017 Jan 30;6(1):e295–. doi: 10.1038/oncsis.2016.86 (PMC5294249; doi:10.1038/oncsis.2016.86)
Supplement: Supplementary Table S1 [file oncsis201686x3.pdf]

Supplementary Table S1. List of primary antibodies used for biochemical or functional analyses

Antigen	Host	Catalog	Vendor	Application
E-cadherin	Rab	sc-7870	Santa Cruz Biotechnology	IB
PARP	Rab	9532	Cell Signalling Technology	IB
Cleaved Caspase-3	Rab	9661	Cell Signalling Technology	IB
Bim	Rab	2933	Cell Signalling Technology	IB
Caspase-8	Ms	9746	Cell Signalling Technology	IB
XIAP	Rab	14334	Cell Signalling Technology	IB
Akt	Rab	9272	Cell Signalling Technology	IB
Phospho-Akt	Rab	9271	Cell Signalling Technology	IB
p44/42 MAPK	Rab	9102	Cell Signalling Technology	IB
Phospho-p44/42 MAPK	Rab	9101	Cell Signalling Technology	IB
N-Myc	Rab	sc-56729	Santa Cruz Biotechnology	IB
c-Myc	Rab	CME 415 AK, CK	BIOCARE MEDICAL	IHC,IB
FAK	Rab	sc-558	Santa Cruz Biotechnology	IHC,IB
p-397-FAK	Rab	sc-11765-R	santa Cruz Biotechnology	IB
β1 integrin	MS	MAB1959	Millipore	FACS
β1 integrin	Rab	AB1952	Millipore	IB
β-actin	Ms	A2228	Sigma-Aldrich	IB
