# Supplementary material for: Inhibition of the integrin/FAK signaling axis and c-Myc synergistically disrupts ovarian cancer malignancy
Source: Oncogenesis. 2017 Jan 30;6(1):e295–. doi: 10.1038/oncsis.2016.86 (PMC5294249; doi:10.1038/oncsis.2016.86)
Supplement: Supplementary Table S2 [file oncsis201686x4.pdf]

Supplementary Table S2. List of small molecule inhibitors used for VS-6063-based screening

Inhibitors		Catelog	Vendor
No.	Name		
1	Nintedanib	N-9077	LC laboratories
2	PTC-209	104M4711V	Sigma-Aldrich
3	R-Ketorolac	Sc-208241	Santa Cruz
4	VS-6063	S7654	Selleckchem
5	PF-00562271	S2672	Selleckchem
6	Imatinib	SML-1027	Sigma-Aldrich
7	SB2016763	1616	TOCRIS
8	ICG-001	540712	Milipore
9	Vincristine	V8879	Sigma-Aldrich
10	JQ1	S7110	Selleckchem
11	DAPT	D5942	Sigma-Aldrich
12	5-AZ'	A3656	Sigma-Aldrich
13	Lapatinib	L-4899	LC laboratories
14	Olaparib	F-4545	AChemBlock
15	LGK974	L974-02	StemRD
16	PD-901	A3013	APExBIO
17	XAV939	X3004	Sigma-Aldrich
18	SP600125	1496	TOCRIS
19	Veliparib	V-4703	TOCRIS
20	SB202190	1264	TOCRIS
21	Y27632	S1049	Selleckchem
22	MG132	S2619	Selleckchem
23	Dorsomorphin	S7306	Selleckchem
24	KU-55933	S1092	Selleckchem
25	LBH589	S1030	Selleckchem
26	ABT-7373	S1002	Selleckchem
27	PP2	P0042	Sigma-Aldrich
28	MK-2206	S1078	Selleckchem
29	Iressa	3000	TOCRIS
30	SCH527123	473727-83-2	Medchem Express
